# 2842. Characteristics and Outcomes of Urinary Tract Infections (UTIs) Caused by Enterococci at a Tertiary Academic Medical Center in Saudi Arabia

**DOI:** 10.1093/ofid/ofad500.2452

**Published:** 2023-11-27

**Authors:** Abrar K Thabit, Khalid F Alharthi, Salem M Baotab, Abdullah M Bankhar, Ahmad J Mahrous

**Affiliations:** King Abdulaziz University, Jeddah, Makkah, Saudi Arabia; King Abdulaziz University, Jeddah, Makkah, Saudi Arabia; King Abdulaziz University, Jeddah, Makkah, Saudi Arabia; King Abdulaziz University, Jeddah, Makkah, Saudi Arabia; Umm Al Qura University, Jeddah, Makkah, Saudi Arabia

## Abstract

**Background:**

Enterococci are Gram-positive coccus bacteria that are normally present in the gastrointestinal tract and ordinarily function commensally with humans. They are generally considered opportunistic and often associated with healthcare-associated infections. We aimed to characterize patients with UTIs due to Enterococci and their outcomes.

**Methods:**

This was a retrospective cohort study between June 2012 and November 2022 of patients who had clinically and microbiologically confirmed Enterococcal UTI. Inclusion criteria included a urine culture positive for *E. faecalis* or *E. faecium* with a count of ≥ 10^5^ CFU/mL and having urinary tract symptoms.

**Results:**

Of 4,094 patients screened, 125 were eligible and included. Patients had a median age of 63.3 years and were mostly females (60%). The most common characteristics were having a catheter, hospitalization in a non-ICU ward, and recent use of antibiotics within 90 days. Infection with *E. faecalis* was more common than *E. faecium* (78.4% vs. 21.6%). However, the latter exhibited higher rates of antibiotic resistance (*P* < 0.001) and was associated with significantly higher median temperature (38 vs. 37.1°C; *P* < 0.001), mortality rates (24% vs. 6.9%; *P* = 0.020), and median length of stay (32 vs. 13 days; *P* < 0.001).

**Conclusion:**

Most patients with enterococcal UTIs had a history of catheter and recent antibiotic use and were mostly females and hospitalized in non-ICU wards. Patients infected with *E. faecium* had more severe episodes compared with patients infected with *E. faecalis*. Therefore, patients infected with *E. faecium* should be followed more closely and have their antibiotics evaluated by infectious diseases experts of physicians and clinical pharmacists to potentially improve their outcomes.

**Disclosures:**

**All Authors**: No reported disclosures

